# Supplementary material for: Tead1a Initiates Transcriptional Priming Through the TEAD1a/YAP‐Notch1‐Spi1/Cebpα Axis to Promote Neutrophil Fate
Source: Adv Sci (Weinh). 2025 Aug 28;12(41):e05441. doi: 10.1002/advs.202505441 (PMC12591127; doi:10.1002/advs.202505441)
Supplement: Supplementary file 4 — Supplementary Dataset [file ADVS-12-e05441-s004.zip › 3-6.zip!/3-6/02-6.cluster/cluster17/cluster17_featureplot.pdf]

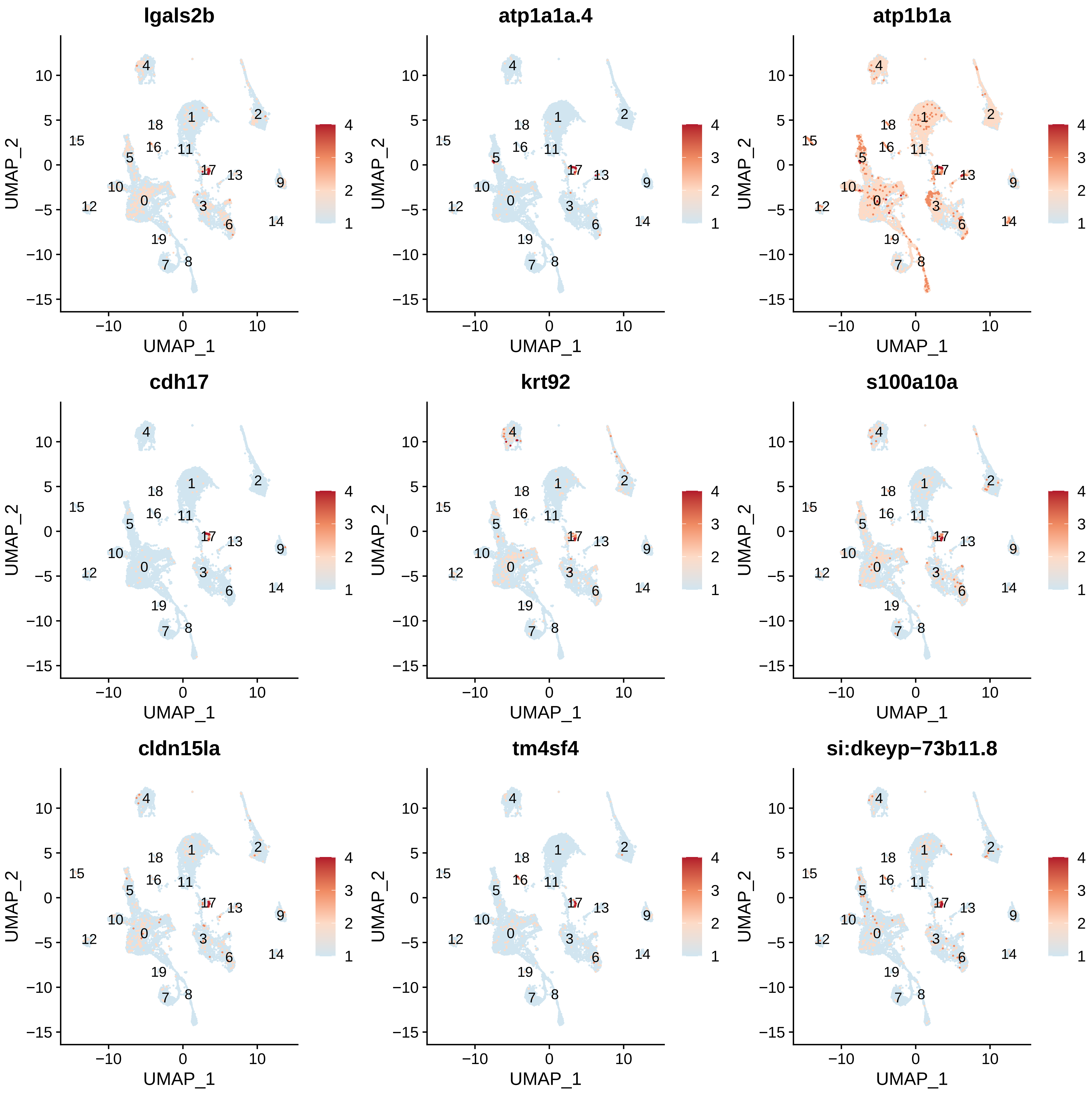

lgals2b
atp1a1a.4
atp1b1a
cdh17
krt92
s100a10a
cldn15la
tm4sf4
si:dkeyp−73b11.8
UMAP_1
UMAP_2
